# A longitudinal inquiry into the vicious cycle of social media addiction and self-injury: the moderating role of resilience

**DOI:** 10.3389/fpsyt.2026.1796643

**Published:** 2026-04-24

**Authors:** Meihong Deng, Wenyi Guo

**Affiliations:** 1 Changsha Ningxiang Psychiatric Hospital, Changsha, Hunan, China; 2 Faculty of Health and Wellness, City University of Macau, Macau, China; 3 Peking University HSBC Business School, Shenzhen, Guangdong, China

**Keywords:** adolescent, cross-lagged panel model, longitudinal study, moderation, non-suicidal self-injury, psychological resilience, social media addiction

## Abstract

**Background:**

The reciprocal relationship between social networking addiction (SNA) and non-suicidal self-injury (NSSI) represents a critical, yet poorly understood, feedback loop in adolescent psychopathology. This study aimed to longitudinally test a “vicious cycle” model, examining the bidirectional effects between SNA and NSSI, and to investigate psychological resilience as a potential protective factor that could disrupt this harmful dynamic.

**Methods:**

A three-wave longitudinal study was conducted with a large cohort of 2,628 Chinese high school students (mean age = 16.1 years; 53.1% female) over a 12-month period. Participants completed measures of SNA, NSSI frequency, and psychological resilience at each wave. A cross-lagged panel model (CLPM) was used to examine the reciprocal, prospective relationships between SNA and NSSI. A multi-group CLPM was then employed to test the moderating role of resilience.

**Results:**

The CLPM revealed significant, positive, and reciprocal cross-lagged effects. SNA at T1 and T2 prospectively predicted increases in NSSI at T2 and T3, respectively (βs = .19 and.17). Conversely, NSSI at T1 and T2 prospectively predicted increases in SNA at T2 and T3 (βs = .14 and.12), providing robust evidence for a vicious cycle. Furthermore, resilience significantly moderated the pathway from SNA to NSSI. For adolescents with low resilience, the effect was strong and significant (β = .25), whereas for those with high resilience, the effect was rendered non-significant (β = .07).

**Conclusions:**

Social networking addiction and non-suicidal self-injury are not merely comorbid but are locked in a mutually reinforcing developmental spiral over time. However, this dangerous cycle is not deterministic. Psychological resilience acts as a powerful protective buffer, effectively uncoupling the link from addictive social media use to self-harm. These findings underscore the urgent need for integrated, dual-focus interventions that address both online and offline maladaptive behaviors, while championing resilience-building as a primary strategy for prevention.

## 1 Introduction

Non-suicidal self-injury (NSSI), the deliberate, self-inflicted destruction of body tissue without conscious suicidal intent, has transitioned from a behavior once confined to the margins of clinical psychiatry to a pervasive and alarming feature of mainstream adolescent experience (1). The sheer scale of this public health issue is captured in stark epidemiological data, with global meta-analyses indicating a lifetime prevalence of approximately 17-18% among adolescents (2, 3). This figure is not an abstract statistic; it represents millions of young people who, in moments of overwhelming distress, turn against their own bodies. In the unique sociocultural context of modern China, where rapid modernization coexists with immense academic pressure, recent studies have documented prevalence rates as high as 28% in community samples of adolescents, signaling an urgent need for culturally-attuned research (4).

From a developmental psychopathology perspective, NSSI is best understood not as a static condition but as a developmental process that typically emerges in early adolescence, a period of profound neurobiological flux and psychosocial challenge (5). The behavior often functions as a brutally effective, albeit maladaptive, coping strategy. Grounded in the Experiential Avoidance Model (EAM), NSSI is conceptualized as a desperate attempt to escape or down-regulate aversive internal states, such as overwhelming sadness, anxiety, or emptiness (6). The sharp physical pain can provide a powerful, momentary distraction from emotional agony, while the sight of blood can offer a tangible validation of internal suffering, culminating in a fleeting sense of relief (7). This powerful negative reinforcement mechanism makes NSSI a particularly insidious and difficult behavior to overcome once it becomes an adolescent’s primary coping tool.

Critically, the developmental trajectory of NSSI is rarely benign. It is a robust predictor of a cascade of future mental health problems and, most ominously, stands as one of the strongest known risk factors for future suicide attempts (8). The Interpersonal-Psychological Theory of Suicide posits that NSSI may serve as a form of painful and provocative practice, eroding the natural fear of death and habituating the individual to physical pain, thereby building the “acquired capability” for suicide (9). This transforms our understanding of NSSI from a “cry for help” to a potential step in the pathway toward lethal self-harm, making the identification of its drivers and moderators a life-or-death imperative.

### 1.1 A vicious cycle of social media addiction and self-injury

As adolescents navigate the turbulent waters of development, the rise of social media has introduced a powerful and unprecedented new current into the risk equation. The digital world has become the primary social ecosystem for many young people, fundamentally reshaping how they interact, form identities, and cope with distress. Within this ecosystem, social networking addiction (SNA)—a behavioral addiction characterized by compulsive, excessive, and dysregulated use of social media platforms—has emerged as a key correlate of poor mental health (10).

Cross-sectional studies have consistently linked SNA with NSSI, yet the nature and directionality of this relationship have remained a critical, unanswered question. Does addictive social media use create the conditions for self-harm? Does the act of self-harm drive adolescents deeper into the online world? Or, as is often the case in developmental psychopathology, do they become locked in a transactional, mutually reinforcing feedback loop? We propose that the latter is most likely, and that this “vicious cycle” can be understood by integrating two complementary theoretical frameworks.

The Interaction of Person-Affect-Cognition-Execution (I-PACE) model offers a compelling framework for understanding how addictive online behaviors can lead to offline harm (11). The model suggests that individuals with certain predisposing vulnerabilities (e.g., negative affectivity, poor emotion regulation) may turn to social media for compensatory reasons—to escape, to seek validation, to feel a sense of belonging. This can initiate a cycle of craving and compulsive use, which in turn has several downstream consequences that elevate NSSI risk. First, it can lead to a distorted view of social reality through the constant exposure to the curated perfection of others, fostering intense social comparison, envy, and feelings of inadequacy—all established precursors to NSSI (12). Second, it increases exposure to cyberbullying and, critically, to online communities and content that normalize or even glorify self-harm, creating a powerful social contagion effect (13). Third, from a neurocognitive perspective, the variable-ratio reinforcement schedule of social media (e.g., unpredictable notifications) can hijack the brain’s reward system, impairing inhibitory control and increasing impulsivity, which are core deficits in both addiction and NSSI (14).

Conversely, the EAM, traditionally applied to NSSI itself, can be extended to explain the pathway from self-harm to problematic social media use. Following an episode of NSSI, adolescents are often flooded with intense secondary emotions, such as shame, guilt, and self-loathing (15). To cope with these painful post-NSSI states, they may retreat into the digital world. Social media can offer a seemingly safe, anonymous, and accessible space to distract from these feelings, to seek solace in online communities, or to find a sense of identity and validation that feels absent in their offline lives (16). This reliance on the online world as a primary means of regulating the emotional aftermath of self-harm can create a powerful dependency, fostering a pattern of compulsive use that meets the criteria for addiction. This creates a pernicious feedback loop: offline distress leads to NSSI, the shame of which leads to escapist and addictive social media use, which in turn generates more distress and risk for future NSSI.

Testing this bidirectional, “vicious cycle” hypothesis requires a longitudinal design that can model these reciprocal effects over time. This is a primary objective of the present study.

### 1.2 The protective shield resilience

While the prospect of a vicious cycle is alarming, it is crucial to recognize that development is not deterministic. A core tenet of developmental psychopathology is the study of individual differences in response to adversity. This raises a fundamental question: Why do some adolescents, even when caught in the pull of problematic social media use, avoid self-harm, while others succumb? The concept of psychological resilience offers a powerful, strengths-based lens for understanding this variability (17).

Resilience is not a static, invulnerable trait, but rather a dynamic and multi-faceted process of positive adaptation in the context of significant adversity (18). It encompasses a range of internal assets and external resources, including cognitive skills (e.g., positive reappraisal, problem-solving), emotional regulation capacities, high self-esteem and self-efficacy, a sense of purpose, and the ability to recruit and maintain social support (19). These very characteristics stand in direct opposition to the core vulnerabilities—such as negative affectivity, poor impulse control, and social disconnection—that are thought to underlie both SNA and NSSI.

According to the classic Stress-Buffering Model, protective factors like resilience can act as a moderator, weakening the pathogenic link between a risk factor (stressor) and a negative outcome (20). In the context of our proposed vicious cycle, resilience is hypothesized to act as a protective shield, buffering the harmful pathway from SNA to NSSI. For an adolescent with high resilience, the negative sequelae of compulsive social media use—such as feelings of inadequacy from social comparison or anxiety from online conflict—may be mitigated by their strong coping skills, stable sense of self-worth, and robust offline social networks. They may be better able to cognitively reappraise negative online experiences, disengage from problematic use, and seek help from trusted adults or peers when needed. In contrast, for an adolescent with low resilience, the pathway from the distress generated by SNA to the act of NSSI may be much more direct and potent, as they lack the internal and external resources to counter the negative impact of their online world.

Investigating the moderating role of resilience is therefore crucial for moving beyond a purely risk-focused model to a more balanced, comprehensive understanding of adolescent mental health. It allows us to identify not only who is most at risk, but also *what* protective mechanisms can be leveraged to design effective, strengths-based interventions. A second primary objective of this study is to empirically test this stress-buffering hypothesis in a longitudinal framework, examining whether resilience can indeed uncouple the vicious cycle between SNA and NSSI over time.

### 1.3 Aims and hypotheses

To address these critical gaps in the literature, the present study employs a three-wave longitudinal design to test a comprehensive model of the dynamic interplay between SNA, NSSI, and psychological resilience in a large, non-Western sample of adolescents. Our study has two primary objectives:

To examine the bidirectional longitudinal relationship between SNA and NSSI. We will use a cross-lagged panel model (CLPM) to rigorously test the “vicious cycle” hypothesis, examining whether SNA predicts future increases in NSSI, and vice versa, over a 12-month period.

To investigate the moderating role of psychological resilience. We will test the stress-buffering hypothesis by examining whether resilience moderates the longitudinal pathways between SNA and NSSI, thereby identifying a key protective factor that can disrupt this harmful cycle.

Based on the theoretical frameworks discussed, we propose the following hypotheses:

Hypothesis 1 (The Vicious Cycle): There will be significant, positive, and reciprocal cross-lagged effects between SNA and NSSI. Specifically, higher levels of SNA at one time point will predict a significant increase in NSSI at the subsequent time point, and higher levels of NSSI at one time point will predict a significant increase in SNA at the subsequent time point.
Hypothesis 2 (The Protective Shield): Psychological resilience will significantly moderate the cross-lagged path from SNA to NSSI. Specifically, the positive association between SNA and subsequent NSSI will be significantly weaker for adolescents with high levels of resilience compared to those with low levels of resilience.

By testing these hypotheses, this study aims to make several important contributions. It will provide one of the first robust, longitudinal tests of the reciprocal relationship between SNA and NSSI, moving beyond the ambiguity of cross-sectional data. It will also be one of the first studies to empirically test the buffering role of resilience in this specific context, providing crucial insights for the development of strengths-based prevention and intervention programs. Finally, by focusing on a large sample of Chinese adolescents, this study will add valuable cultural diversity to a literature that remains predominantly Western-centric, testing the generalizability of these psychological processes.

## 2 Methods

### 2.1 Participants and procedure

This study utilized a three-wave longitudinal design. The sample was drawn from ten public high schools in Hunan Province, a large and economically diverse province in Central China. A multi-stage cluster sampling method was employed. From a total pool of 180 classes across the ten schools, 60 classes (6 per school) were randomly selected using a computer-generated sequence. Class sizes ranged from 42 to 55 students (M = 48.5, SD = 3.2). First, schools were purposively selected to represent a mix of urban, suburban, and rural settings, as well as varying levels of academic prestige. Second, within each selected school, several intact classes from each grade level (10th, 11th, and 12th) were randomly selected for participation.

The study protocol received full ethical approval from the Institutional Review Board (IRB) of City University of Macau (IRB Ref: FHW-ER-2425-116). Prior to the first wave of data collection, comprehensive information sheets were distributed to all prospective participants and their legal guardians, The study was presented to students and guardians as a longitudinal investigation into “Adolescent Lifestyle and Emotional Health,”aiming to understand the interplay between digital media habits, psychological strengths, and emotional well-being. outlining the study’s aims, procedures, voluntary nature, and strict confidentiality protocols. Active written informed consent was obtained from a legal guardian for every participant, and all participating adolescents provided their own written assent.

Data were collected at three time points, each separated by a six-month interval: Wave 1 (T1; baseline), Wave 2 (T2; 6 months post-baseline), and Wave 3 (T3; 12 months post-baseline). The final sample (N = 2,628) consisted of 1,233 males (46.9%) and 1,395 females (53.1%), with ages ranging from 14 to 19 years. Regarding residence, 58.4% (n = 1,535) were from urban areas and 41.6% (n = 1,093) from rural areas. SES scores were normally distributed (Range: -2.85 to 2.42). The study achieved high retention rates, with 2,415 participants (91.9%) completing the T2 survey and 2,339 (89.0%) completing the T3 survey.

To ensure a standardized and private data collection environment, all surveys were administered online via a secure platform during designated class periods in school computer labs. Trained research assistants were present to supervise the sessions, answer any procedural questions, and ensure participant confidentiality. Participants were informed that their responses were anonymous and would have no bearing on their academic standing. As a small token of appreciation for their time and effort, a modest monetary compensation was provided to each participant at each wave.

To assess the potential for attrition bias, we conducted a series of t-tests and chi-square tests comparing participants who completed all three waves with those who dropped out at T2 or T3. These analyses revealed no significant differences between the groups on key baseline demographic variables (age, gender, family income, parental education) or the main study variables (NSSI, SNA, resilience). This suggests that attrition was likely missing at random (MAR), allowing us to use Full Information Maximum Likelihood (FIML) estimation in our subsequent analyses. FIML is a state-of-the-art technique for handling missing data that utilizes all available information from all participants to produce unbiased parameter estimates, thereby maximizing statistical power and reducing bias (21).

### 2.2 Measures

All measures were administered in their validated Mandarin Chinese versions. For scales originally developed in English, standard back-translation procedures were rigorously followed to ensure linguistic and conceptual equivalence (22). All multi-item scales were assessed for internal consistency using Cronbach’s alpha at each wave, and all demonstrated good to excellent reliability.

Social Networking Addiction (SNA):SNA was assessed using the Social Networking Addiction Scale for Adolescents (SNASA; 23), a 24-item scale specifically developed and validated for use with Chinese adolescents. The scale is grounded in the core components of behavioral addiction (24) and assesses six dimensions: salience, mood modification, tolerance, withdrawal, conflict, and relapse. A sample item is, “How often have you felt an urge to use social media more and more?” Participants rated each item on a 5-point Likert scale from 1 (never) to 5 (always). A total score was calculated by summing the items, with higher scores indicating greater levels of addictive social media use. Previous studies have reported strong convergent validity with the Bergen Social Media Addiction Scale (r = .78) and a two-week test-retest reliability of.86. The scale demonstrated excellent internal consistency in the present study across all three waves (α = .91,.92, and.93 at T1, T2, and T3, respectively).

Non-Suicidal Self-Injury (NSSI):The frequency of NSSI was measured using a 12-item checklist adapted from the widely used Functional Assessment of Self-Mutilation (FASM; 25). Participants were first presented with a clear definition of NSSI, explicitly distinguishing it from suicidal behavior and accidental injuries. They were then asked to indicate how many times in the preceding six months they had intentionally engaged in specific self-harm behaviors (e.g., cutting, burning, severe scratching, hitting oneself). Response options were on a 5-point ordinal scale: 0 (never), 1 (once), 2 (2–3 times), 3 (4–5 times), or 4 (more than 5 times). A total frequency score was created by summing the responses across the 12 items. It has shown high agreement with clinical interviews (κ = .82) and stable 1-month test-retest reliability (r = .80). This measure has been used extensively in research with Chinese adolescents and has shown good psychometric properties. The internal consistency in this study was excellent (α = .89,.90, and.89 at T1, T2, and T3, respectively).

Psychological Resilience:Resilience was measured using the 10-item Connor-Davidson Resilience Scale (CD-RISC-10; 26), one of the most robust and widely used measures of resilience. The scale assesses the ability to cope with stress and bounce back from adversity. Sample items include, “Able to adapt to change,” and “Can deal with whatever comes my way.” Participants rated each item on a 5-point scale from 0 (not true at all) to 4 (true nearly all the time). Its construct validity is well-established via factor analysis in Chinese samples, with test-retest reliability reported at.84. The scale has been validated in Chinese samples and demonstrated excellent internal consistency in our study (α = .93,.94, and.94 at T1, T2, and T3, respectively). To ensure the construct was being measured consistently over time—a critical prerequisite for longitudinal moderation analysis—we conducted formal tests of measurement invariance. The results supported strict invariance across the three waves, indicating that the scale functioned identically at each time point.

Control Variables: To isolate the unique relationships between our variables of interest, we controlled for several potential demographic confounders in all our models. These included age (in continuous years), gender (0 = male, 1 = female), Urban and rural residences were defined according to the National Bureau of Statistics of China’s administrative classifications. place of residence (0 = rural, 1 = urban), and a composite measure of socioeconomic status (SES). SES was calculated as a composite index by standardizing and averaging paternal and maternal education levels and annual family income.

### 2.3 Data analysis strategy

All statistical analyses were conducted using Mplus 8.7 (27), a powerful software package for latent variable and structural equation modeling (SEM). The analysis proceeded in three main steps.

Step 1: Preliminary Analyses. We first calculated descriptive statistics (means, standard deviations, frequencies) and bivariate correlations for all study variables at T1. This provided an initial overview of the data and the cross-sectional relationships between the key constructs, and allowed us to check for any potential issues with multicollinearity.

Step 2: Testing the Vicious Cycle (Hypothesis 1). To test the hypothesis of a bidirectional relationship, we specified a cross-lagged panel model (CLPM). The CLPM is the state-of-the-art approach for examining reciprocal, prospective effects between two or more variables over time (28). The model was specified to include:

Autoregressive paths: These paths (e.g., from T1 NSSI to T2 NSSI) estimate the stability or carry-over effect of each variable on itself over time.

Cross-lagged paths: These are the key paths for testing our hypothesis. They estimate the prospective effect of one variable on the other at the subsequent time point (e.g., from T1 SNA to T2 NSSI, and from T1 NSSI to T2 SNA), after controlling for the stability of the outcome variable.

Covariances: Within-wave correlations between the residuals of SNA and NSSI were estimated at each time point to account for shared variance not explained by the model’s predictors.

All demographic covariates were included in the model, with paths to both SNA and NSSI at all three waves to control for their influence.

Step 3: Testing the Protective Shield (Hypothesis 2). To test the moderating role of resilience, we used a multi-group CLPM approach, a robust method for testing moderation in a longitudinal SEM context. We first created three groups based on participants’ T1 resilience scores: a low resilience group (scores ≤ 1 SD below the mean; n = 418), a medium resilience group (scores between -1 SD and +1 SD; n = 1792), and a high resilience group (scores ≥ 1 SD above the mean; n = 418). We then ran the CLPM simultaneously for all three groups. To formally test for moderation, we compared the fit of a constrained model, where the key cross-lagged path from SNA to NSSI was forced to be equal across the three groups, to an unconstrained model where this path was allowed to vary freely. A significant degradation in model fit (indicated by a significant chi-square difference test, Δχ²) would provide evidence for moderation. We then examined the specific path coefficients in each group to interpret the nature of the moderation effect.

Model Fit: For all SEM models, we assessed model fit using a combination of well-established fit indices: the chi-square test (χ²), the Comparative Fit Index (CFI), the Tucker-Lewis Index (TLI), the Root Mean Square Error of Approximation (RMSEA), and the Standardized Root Mean Square Residual (SRMR). Following the widely accepted guidelines proposed by Hu and Bentler (29), good model fit is indicated by CFI and TLI values >.95, RMSEA <.06, and SRMR <.08.

## 3 Results

### 3.1 Preliminary analyses

Descriptive statistics for all study variables and demographic characteristics at each of the three waves are presented in **Table 1**. The sample included 1,233 males (46.9%) and 1,395 females (53.1%), with ages ranging from 14 to 18 years. Regarding residence, 1,114 (42.4%) were from urban areas and 1,514 (57.6%) from rural areas. SES scores were standardized with a mean of 0 and ranged from -2.85 to 2.42. At baseline (T1), the prevalence of NSSI was substantial, with 27.8% of adolescents reporting at least one episode in the past six months. The mean scores for the key variables were as follows: NSSI frequency (M = 1.95, SD = 2.50), SNA (M = 29.1, SD = 10.5), and psychological resilience (M = 30.5, SD = 6.2). A repeated measures ANOVA revealed a small but statistically significant increase in SNA over the 12-month period, F(2, 5254) = 4.51, p <.05, ηp² = .002. In contrast, mean levels of NSSI showed a slight, non-significant decrease, and psychological resilience remained highly stable. The bivariate correlations between all variables at T1 were consistent with theoretical expectations. Notably, resilience was significantly and negatively correlated with both NSSI (r = -.35, p <.001) and SNA (r = -.28, p <.001), providing initial cross-sectional support for its protective role.

**Table 1:** Descriptive Statistics and Inter-correlations at T1.

Variable	M	SD	1	2	3
Gender (Female %)	53.1%	–	–	–	–
Age (years)	16.1	1.2	–	–	–
Residence(Urban %)	42.4%	–	–	–	–
SES	0	1.0	–	–	–
1. NSSI (T1)	1.95	2.5	–		
2. SNA (T1)	29.1	10.5	.41**	–	
3. Resilience(T1)	30.5	6.2	-.35**	-.28**	–
4. NSSI(T2)	1.89	2.45	.42**	.39**	-.32**
5. SNA (T2)	29.85	10.7	.38**	.58**	-.27**
6. Resilience(T2)	30.55	6.25	-.33**	-.26**	.65**
7. NSSI (T3)	1.85	2.4	.35**	.33**	-.30**
8. SNA (T3)	30.2	10.8	.32**	.55**	-.25**
9. Resilience(T3)	30.6	6.3	-.30**	-.25**	.63**

The symbol ** indicates that the correlation coefficient is statistically significant at the p < .01 level (two-tailed).

### 3.2 Main analysis: the vicious cycle cross-lagged panel model

**Figure 1:**
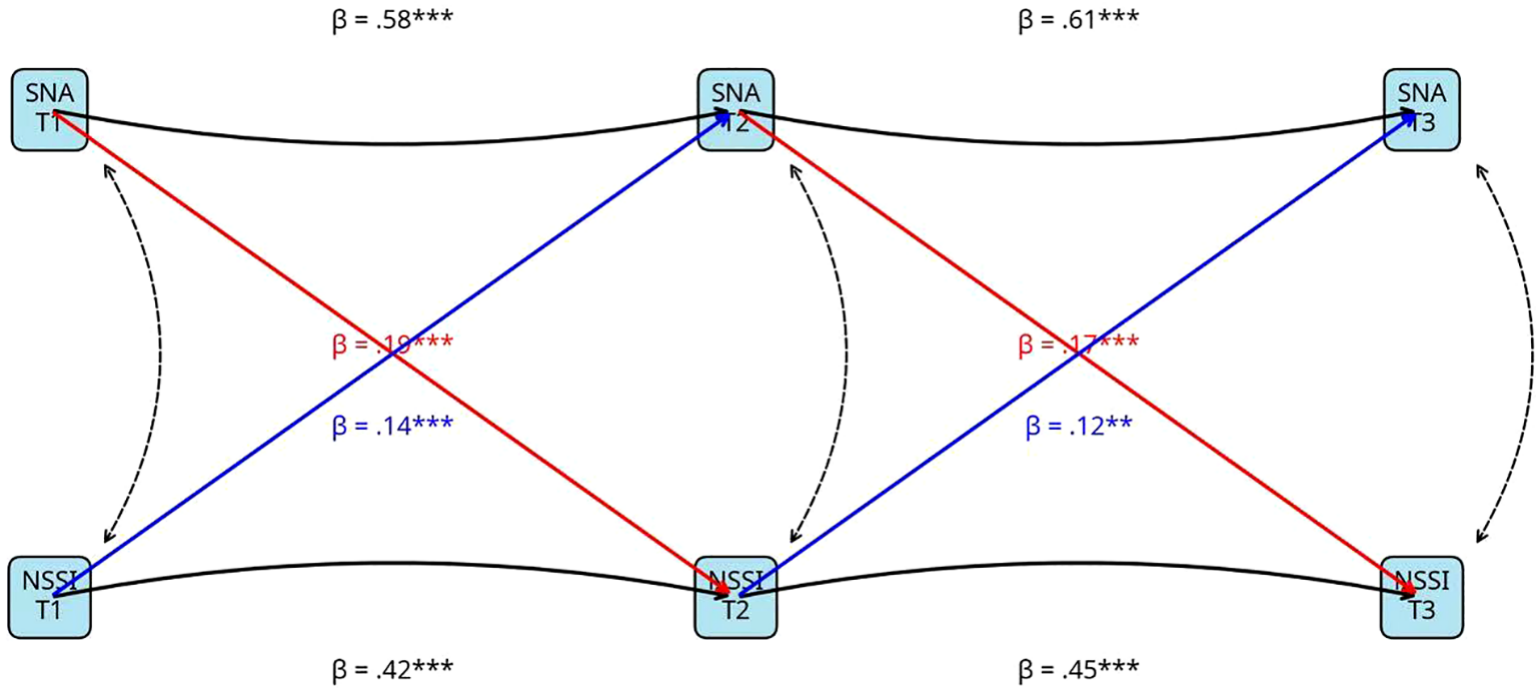
Cross-lagged panel model of SNA and NSSI. The symbol *** indicates that the standardized path coefficient (β) is statistically significant at the p < .001 level. Additionally, the symbol ** (appearing on one path, e.g., β = .12**) indicates significance at the p < .01 level.

To test Hypothesis 1, we estimated the CLPM. The model provided an excellent fit to the data: χ²(34) = 75.8, p <.001; CFI = .992; TLI = .987; RMSEA = .024 (90% CI = [.018,.031]); SRMR = .019. The standardized path coefficients for the key model paths are depicted in **Figure 1**.

Stability Paths (Autoregressive Effects). Both SNA and NSSI demonstrated substantial stability over the 12-month period. The autoregressive paths for SNA were strong and significant from T1 to T2 (β = .58, p <.001) and from T2 to T3 (β = .61, p <.001). Similarly, the autoregressive paths for NSSI were also significant and moderate in magnitude from T1 to T2 (β = .42, p <.001) and from T2 to T3 (β = .45, p <.001). These findings indicate that both behaviors are relatively stable, trait-like phenomena in this adolescent sample.

Cross-Lagged Paths. In strong support of Hypothesis 1, we found significant, positive, and reciprocal cross-lagged effects across both time lags.

Path from SNA to NSSI: Higher levels of SNA at T1 significantly predicted an increase in NSSI at T2 (β = .19, p <.001). This prospective effect was replicated from T2 to T3, where T2 SNA significantly predicted an increase in T3 NSSI (β = .17, p <.001).

Path from NSSI to SNA: Conversely, higher levels of NSSI at T1 significantly predicted an increase in SNA at T2 (β = .14, p <.001). This reciprocal effect was also replicated from T2 to T3 (β = .12, p <.01).

These results provide robust longitudinal evidence for a bidirectional, mutually reinforcing relationship between SNA and NSSI over time, confirming the “vicious cycle” hypothesis.

### 3.3 Moderation analysis: the protective shield of resilience

To test Hypothesis 2, we compared the fit of the multi-group CLPM with the cross-lagged path from SNA to NSSI constrained to be equal across the three resilience groups versus an unconstrained model where this path was freely estimated. The chi-square difference test was significant (Δχ²(2) = 15.4, p <.001), indicating that the strength of the path from SNA to NSSI differed significantly across the resilience groups, thus providing strong support for the moderation hypothesis.

**Figure 2:**
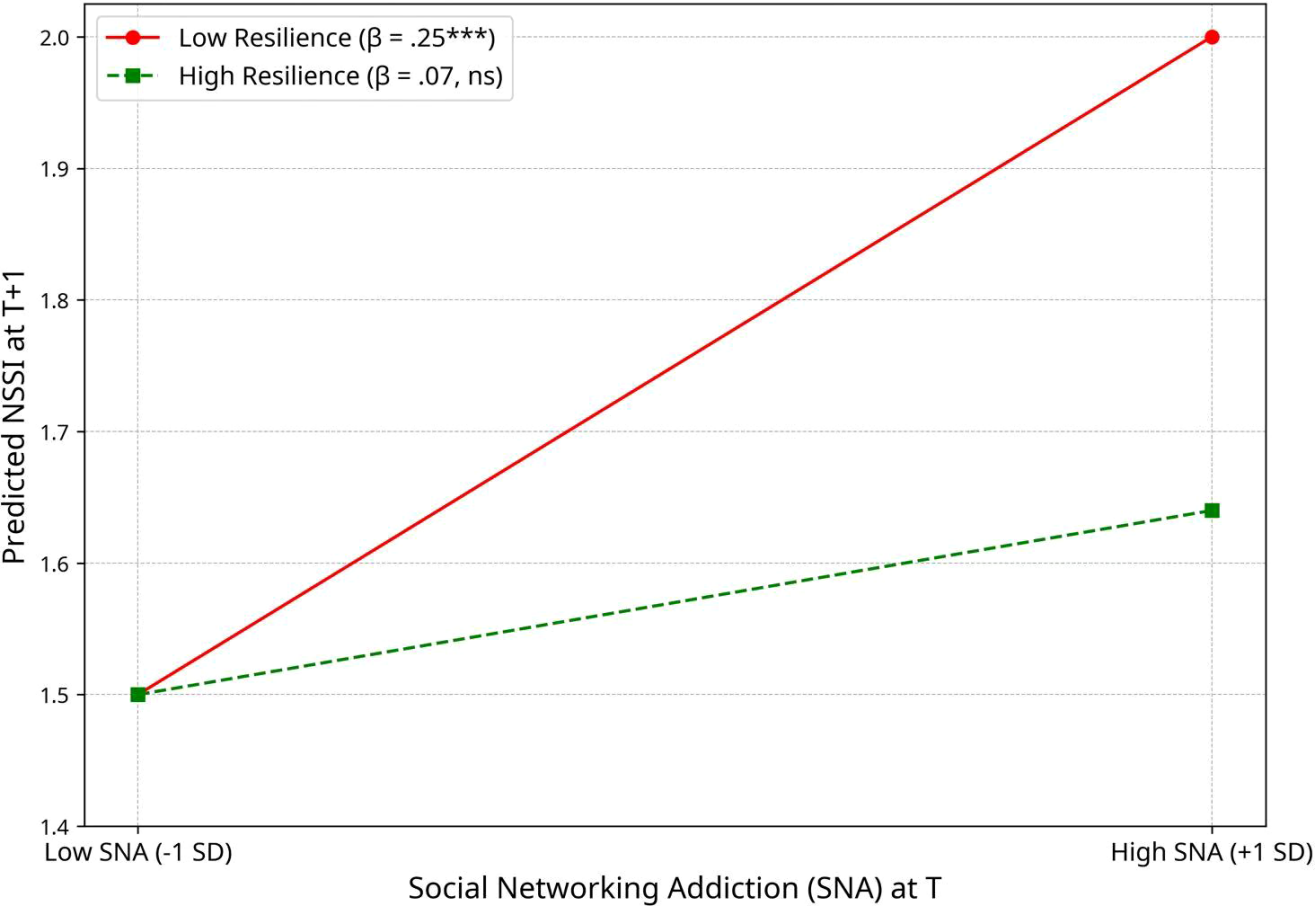
Moderating effect of resilience on the SNA -> NSSI pathway.

As shown in **Figure 2**, the prospective effect of SNA on NSSI was strongest and highly significant in the low resilience group (T1-T2: β = .25, p <.001; T2-T3: β = .23, p <.001). The effect was weaker but still significant in the medium resilience group (T1-T2: β = .18, p <.001; T2-T3: β = .16, p <.001). Most notably, in the high resilience group, the path from SNA to NSSI was rendered non-significant at both time lags (T1-T2: β = .07, p = .15; T2-T3: β = .06, p = .21).

These findings provide powerful support for the stress-buffering model. High psychological resilience appears to act as a protective shield, effectively decoupling the harmful longitudinal link between social networking addiction and subsequent engagement in non-suicidal self-injury.

## 4 Discussion

This study, using a three-wave longitudinal design with a large adolescent sample, provides one of the first and most robust examinations of the dynamic interplay between social networking addiction, non-suicidal self-injury, and psychological resilience. Two major findings emerged that significantly advance our understanding of adolescent mental health in the digital age, with profound implications for theory, clinical practice, and public health policy.

### 4.1 The vicious cycle of SNA and NSSI

Our first major finding provides compelling longitudinal evidence for a “vicious cycle”—a mutually reinforcing, bidirectional relationship between SNA and NSSI. As hypothesized, not only did addictive social media use prospectively predict increases in self-harm, but self-harm also prospectively predicted increases in addictive social media use over a 12-month period. This moves our understanding beyond a simple, unidirectional risk model to a more complex and dynamic transactional framework (30). It suggests that these two behaviors are not merely comorbid; they are locked in a pernicious developmental feedback loop, where online and offline distress fuel each other over time.

The pathway from SNA to NSSI confirms the tenets of the I-PACE model and other theories that posit the internet as a risk environment (11). The constant social comparison, the fear of missing out (FoMO), the exposure to cyberbullying, and the algorithmic amplification of harmful content can create a toxic digital milieu that exacerbates underlying vulnerabilities, leading to the emotional dysregulation that precipitates NSSI. Our findings demonstrate that this is not just a concurrent correlation; it is a predictive, causal pathway over time.

Perhaps more novel and clinically crucial is the confirmation of the reverse pathway: NSSI prospectively predicts increases in SNA. This finding lends strong support to an extended version of the Experiential Avoidance Model (6). The act of self-harm, while providing momentary relief, is often followed by a wave of intense negative self-evaluation—shame, guilt, and self-loathing. In the face of these painful emotions, the digital world, with its promise of distraction, anonymity, and connection, becomes an incredibly alluring escape. This retreat into the online world, however, is a Faustian bargain. It provides short-term relief at the cost of long-term dependency, deepening the addictive patterns that, as our model shows, will only increase the likelihood of future self-harm. This transactional model has profound implications. It suggests that clinicians cannot treat NSSI in a vacuum. Simply addressing the act of self-harm without also addressing the adolescent’s relationship with technology is likely to be insufficient, as the underlying function of the behavior (experiential avoidance) will simply be transferred to the digital realm, perpetuating the cycle.

### 4.2 Resilience as a developmental switch

The second, and arguably most hopeful, finding of this study is the powerful moderating effect of psychological resilience. Our results demonstrate that resilience acts as a protective shield, effectively severing the longitudinal link between SNA and the progression of NSSI. For adolescents with high levels of resilience, even if they experienced periods of problematic social media use, it did not translate into an escalation of self-harm. In stark contrast, for those with low resilience, the link was potent and direct. This provides strong empirical support for a classic diathesis-stress model, where SNA acts as a potent stressor that is most likely to trigger the NSSI diathesis in individuals with the pre-existing vulnerability of low resilience (31).

This finding has transformative implications for prevention and intervention. It suggests that the most effective strategy for protecting adolescents from the harms of the digital world may not be to simply try to eliminate the risks, but to build the internal strengths that enable them to navigate those risks successfully. Resilience is not an abstract concept; it is a collection of teachable skills—cognitive reappraisal, problem-solving, emotional literacy, self-efficacy, and the ability to seek and receive social support (32). Evidence from meta-analyses (e.g., 33, 34) confirms that resilience is a malleable construct that can be significantly enhanced through structured school-based interventions, such as Cognitive Behavioral Therapy (CBT) and mindfulness-based resilience training. Our findings provide a strong empirical mandate for shifting the focus of public mental health efforts from a purely deficit-focused model to a strengths-based, preventative one. Fostering resilience can be conceptualized as a form of “psychological vaccination,” providing adolescents with the internal resources to resist a wide range of psychosocial pathogens, including the addictive pull of social media.

### 4.3 Deeper theoretical integration

Our findings can be further enriched by considering them through the lens of developmental affective neuroscience. The adolescent brain is famously characterized by a maturational gap: the limbic system, responsible for emotional and reward processing, is fully developed and hyper-responsive, while the prefrontal cortex (PFC), which governs top-down regulation and impulse control, is still undergoing protracted maturation (35). This creates a natural period of heightened vulnerability to emotional dysregulation and impulsive behaviors.

The vicious cycle we identified can be seen as a behavioral manifestation of this neurobiological state. The intense emotional distress that can trigger both NSSI and escapist social media use is processed by the hyper-reactive limbic system. Both behaviors—the physical relief of NSSI and the social validation of SNA—provide a powerful, immediate reward signal via the dopamine system, strongly reinforcing the behavior (36). The underdeveloped PFC struggles to inhibit these prepotent, reward-driven impulses. The reciprocal relationship we observed suggests that engaging in one behavior (e.g., NSSI) may prime the neural circuits for the other (e.g., SNA) by further sensitizing the reward system and depleting limited cognitive control resources.

Resilience, from this perspective, can be conceptualized as the behavioral output of a more mature and better-integrated neural architecture. The cognitive skills central to resilience—such as positive reappraisal and problem-solving—are functions of the PFC. A more resilient adolescent may have stronger functional connectivity between their PFC and limbic structures, allowing for more effective top-down regulation of emotional impulses (37). When faced with the urge to escape distress via SNA or NSSI, a resilient adolescent’s better-developed PFC can intervene, inhibiting the impulse and activating more adaptive, long-term strategies. Our finding that resilience severs the link between SNA and NSSI provides compelling behavioral evidence for this neurodevelopmental process of successful self-regulation.

### 4.4 Clinical and public health implications

The finding of a vicious cycle demands that clinicians working with adolescents presenting with either NSSI or problematic internet use must routinely screen for the other. Treatment should be integrated. For example, Dialectical Behavior Therapy (DBT), a gold-standard treatment for NSSI, could be adapted to include modules on mindful technology use, coping with online triggers (like social comparison or cyberbullying), and building healthy offline social connections to reduce reliance on online validation. Therapy should not only focus on reducing symptoms but also on actively building the client’s resilience. This could involve techniques from Positive Psychology or Acceptance and Commitment Therapy (ACT) to help adolescents identify their values, build self-efficacy, and practice cognitive flexibility. The goal is to equip them with a toolkit of adaptive coping strategies that are more effective and less harmful than NSSI or digital escape.

The most impactful implication is the need for universal, school-based programs focused on social-emotional learning (SEL) and resilience-building. These programs should be a core part of the curriculum, not an optional add-on. Teaching all students the skills of emotional regulation, cognitive reappraisal, and healthy relationship-building is a cost-effective, upstream approach that can inoculate a generation against a wide range of mental health problems. Digital literacy education must evolve beyond simplistic warnings about “screen time.” It needs to become a form of psychological self-defense training for the digital age. Curricula should teach adolescents to be critical consumers of media, to understand the psychological mechanisms of addictive design, to manage social comparison, and to cultivate a healthy balance between their online and offline identities. While building individual resilience is crucial, we must also address the environmental factors that make the digital world so toxic for many. This includes advocating for policies that hold tech companies accountable for the addictive design of their platforms and that push for design changes that prioritize user well-being over engagement at all costs.

While this study has significant strengths, a rigorous and transparent discussion of its limitations is essential for contextualizing the findings and guiding future research. Although we used validated scales, self-report measures for constructs like SNA and NSSI have inherent limitations. For SNA, self-report may not accurately capture the true amount of time spent on social media, and it can be influenced by social desirability bias. Future studies could triangulate self-report data with more objective measures, such as data passively collected from participants’ smartphones (with their explicit consent), to provide a more accurate picture of their digital behavior (38). For NSSI, while self-report is the standard, underreporting due to shame and stigma is a persistent concern. Moreover, our frequency measure, while common, does not capture the severity, methods, or functions of NSSI, all of which are clinically important variables. Future research could employ Ecological Momentary Assessment (EMA), using smartphone apps to prompt participants for in-the-moment reports of their emotions, urges, and behaviors. This would provide a much more granular, real-time understanding of the vicious cycle as it unfolds in daily life.

The CLPM, while a powerful tool for examining reciprocal effects, simplifies a highly complex reality. It assumes that the relationships between variables are linear and homogenous across the population. It is possible that there are non-linear dynamics at play or that there are distinct subgroups of adolescents with different developmental trajectories. Future research could use more advanced statistical techniques, such as Latent Growth Mixture Modeling, to identify these heterogeneous trajectories and their unique predictors. Furthermore, as noted, our model does not capture the full transactional nature of development, where an adolescent’s behavior also influences their environment. A dynamic systems or network analysis approach could be a fruitful avenue for modeling these complex feedback loops in future studies.

A major strength of this study is its large, non-Western sample. However, this also represents a limitation in terms of generalizability. The cultural context of China with its emphasis on collectivism, family harmony, and intense academic pressure, it likely shapes the expression of distress and the role of social media in unique ways. For example, the pressure to uphold family honor may make the shame associated with NSSI particularly acute, potentially driving a stronger need for the anonymity of the online world. The findings, therefore, need to be replicated in diverse cultural contexts to distinguish the universal psychological processes from their culturally-specific manifestations. Cross-cultural comparative studies are a critical next step for the field.

Our study treated resilience as a global construct. While this is a common and valid approach, it masks the complexity of this multi-faceted construct. Resilience is comprised of numerous cognitive, emotional, and social components. Is it cognitive flexibility that is most protective? Or is it the ability to recruit and maintain social support? Or perhaps a strong sense of self-efficacy? Future research should use a component-based approach to resilience, measuring its different facets and examining which ones are most effective at buffering the SNA-NSSI link. This would allow for the development of more targeted and efficient resilience-building interventions, focusing on the specific skills that provide the most protective benefit.

## 5 Discussion

### 5.1 Mechanisms and Implications

The confirmation of a reciprocal relationship between SNA and NSSI is the cornerstone of this study, providing a longitudinal anchor for a transactional model of digital-age psychopathology. This finding compels us to move beyond linear, unidirectional thinking and to embrace the complexity of developmental feedback loops. Several specific mechanisms likely contribute to this cycle.

The cycle may be perpetuated by a shared set of cognitive distortions. Both SNA and NSSI are associated with a preference for immediate over delayed gratification, a focus on short-term emotional relief at the expense of long-term consequences, and a tendency towards ruminative thinking (11, 39). For example, an adolescent might ruminate on a negative social interaction, leading to distress that triggers an urge to self-harm. Following the act, they may ruminate on their feelings of shame, which then triggers an urge to escape into the immersive world of social media. The cognitive habits that sustain one behavior readily transfer to the other.

At its core, the cycle is driven by a fundamental deficit in emotion regulation. The adolescent is caught between two maladaptive strategies for managing unbearable affect: an “approach-based” strategy (SNA, used to seek distraction, validation, or connection) and an “avoidance-based” strategy (NSSI, used to numb or punish). The failure of one strategy may increase the appeal of the other. When social media fails to provide the desired relief or, worse, generates more distress (e.g., through social comparison), the adolescent may feel a sense of hopelessness that leads them to the more potent and immediate relief of NSSI. This suggests that the core target for intervention is not the behaviors themselves, but the underlying inability to tolerate and skillfully manage negative emotions.

The social environment, both online and offline, plays a crucial role. An adolescent who engages in NSSI may feel increasingly isolated from their offline peers due to the secrecy and shame surrounding the behavior. This social isolation can make the seemingly accessible and non-judgmental communities of the online world even more attractive, deepening their reliance on social media. However, within these online spaces, they may be more likely to encounter pro-NSSI content and form connections with others who also self-harm, which can normalize the behavior and even create a sense of identity around it, thus increasing the likelihood of future NSSI (40). This creates a feedback loop where offline isolation drives online immersion, which in turn reinforces the very behavior that contributes to the offline isolation.

### 5.2 Resilience revisited

The finding that resilience buffers the SNA-NSSI pathway is profoundly important, but it is crucial to consider the specific facets of resilience that might be at play.

This involves cognitive flexibility and reappraisal skills. A cognitively resilient adolescent, when faced with a negative online experience (e.g., a critical comment), may be able to reframe it in a less personal and catastrophic way (“This person is just having a bad day”) rather than internalizing it (“I am worthless”). This ability to challenge and modify negative automatic thoughts is a core skill taught in CBT and is likely a key component of the protective effect we observed.

This refers to the ability to effectively regulate one’s emotional responses. A resilient adolescent may have a wider repertoire of healthy coping strategies to draw upon when distressed. Instead of reflexively turning to social media or self-harm, they might go for a run, talk to a friend, or engage in a hobby. Their emotional regulation “toolkit” is more diverse and adaptive.

This involves the ability to build and maintain strong, supportive offline relationships. An adolescent with high social resilience has a robust social safety net. When they feel distressed, they have trusted friends, family members, or mentors to turn to for support. This reduces their dependency on the often-unreliable and superficial validation offered by social media and provides a powerful buffer against feelings of isolation and hopelessness.

It is likely that these facets of resilience work in concert. A resilient adolescent has the cognitive skills to interpret events in a less threatening way, the emotional skills to manage the distress that does arise, and the social skills to seek support when needed. This multi-faceted defense system is what allows them to navigate the challenges of the digital world without succumbing to its most harmful potential. This implies that resilience-building interventions should be comprehensive, targeting all three of these domains.

### 5.3 Final synthesis and conclusion

In conclusion, this study provides a compelling and sobering look into the developmental entanglement of social media and self-harm among adolescents. By providing robust longitudinal evidence for a vicious cycle, we demonstrate that social networking addiction and non-suicidal self-injury are not just comorbid problems but are locked in a dynamic, mutually reinforcing relationship over time. It paints a picture of a developmental spiral where online and offline pain fuel each other. It is a cycle that, left unchecked, can have devastating consequences for an entire generation.

Yet, our findings also provide a powerful and unequivocal message of hope. This cycle is not deterministic. The human capacity for resilience is a potent developmental force, capable of acting as a protective shield that can sever this harmful link. The discovery that high resilience renders the pathogenic pathway from addictive social media use to self-harm non-significant is a profound testament to the power of internal strengths. It suggests that the most promising path forward is not to simply shield adolescents from the risks of the world, but to equip them with the psychological tools they need to navigate those risks successfully.

The implications are clear. We must move towards a dual-pronged approach: one that involves integrated interventions targeting the intertwined nature of online and offline maladaptive behaviors, and another that champions the universal, proactive promotion of psychological resilience. For clinicians, this means looking beyond the presenting problem and assessing the whole ecosystem of an adolescent’s life, online and off. For educators and policymakers, it means recognizing that social-emotional learning and resilience-building are not soft skills, but essential components of a 21st-century education and a core public health priority. By embracing this balanced, strengths-based perspective, we can begin to break the cycle of online and offline suffering and help the digital generation not just to survive, but to thrive.

## Data Availability

The original contributions presented in the study are included in the article/supplementary material. Further inquiries can be directed to the corresponding authors.
